# P074 The falling patient with orthostatic hypotension: an atypical presentation of giant cell arteritis

**DOI:** 10.1093/rap/rkag113.104

**Published:** 2026-11-03

**Authors:** Mohammed Shugaa Addin, Rehan Asif

**Affiliations:** Aneurin Bevan University Health Board, Newport, United Kingdom; Aneurin Bevan University Health Board, Newport, United Kingdom

## Abstract

**Introduction:**

Giant cell arteritis (GCA) is rarely considered in elderly patients presenting with recurrent falls and postural hypotension. This case of an 83 years old-man with recurrent falls, dizziness secondary to postural hypotension, without cranial GCA features and only minimal elevation of inflammatory markers.

In large-vessel vasculitis (LVV), inflammation may impair cerebral perfusion including the carotid and subclavian arteries leading to orthostatic cerebral hypoperfusion, presenting with dizziness and falls.

Extensive investigations initially excluded malignancy. Ultimately, Fluorodeoxyglucose Positron emission tomography – Computed Tomography (FDG PET-CT) showed widespread large-vessel uptake with polymyalgia rheumatica-pattern, confirming GCA and highlighting an important atypical presentation.

**Case description:**

An 83-year-old man was referred with several months of tiredness, weight loss and frequent falls with background of atrial fibrillation and type 2 diabetes. Initial blood tests in primary care showed modestly raised inflammatory markers (CRP 33 mg/L). CT torso (February 2025) in primary care excluded malignancy. His GP commenced prednisolone 15 mg for possible PMR, with limited benefit, PMR was considered unlikely therefore steroids were discontinued. Over subsequent months, CRP remained mildly elevated (24–28 mg/L).

On first review in rheumatology clinic, his main symptom was of worsening postural dizziness and recurrent falls without any temporal headaches, jaw claudication, visual disturbance or clear PMR features. Examination revealed weight 57 kg, normal temporal arteries with no carotid bruits. He had a clear postural drop of 20 mmHg systolic, consistent with postural hypotension. Alternative causes were excluded.

Given persistent symptoms with no alternative explanation of his inflammatory markers, PET-CT was arranged.

The PET-CT (December 2025) demonstrated moderately active LVV, with FDG uptake throughout the aortic arch, descending thoracic and abdominal aorta, origins of the coeliac trunk, both subclavian, proximal carotid and low-grade uptake up to the femoral arteries. PMR-pattern was present without temporal artery involvement or malignancy.

Prednisolone 40 mg daily was initiated. The CRP normalised rapidly, although his symptoms initially persisted. Leflunomide was started early as a steroid sparing agent given impaired glycaemic control and steroid toxicity concerns. Steroids were gradually tapered in line with British Society of Rheumatology (BSR) guidelines.

At 4 months post steroid initiation, his CRP remained normal and symptoms gradually improved with no further falls and complete resolution of postural hypotension. Prednisolone was tapered to 5 mg daily, and his weight increased to 59.4 kg. He remained clinically stable on leflunomide 10 mg with ongoing monitoring.

**Discussion:**

This case illustrates the diagnostic challenges posed by LVV in older adults with comorbidities and atypical symptomatology. The patient lacked classical cranial GCA features and had minimal PMR symptoms, while inflammatory markers were only modestly raised. Early weight loss and anorexia prompted malignancy screening, and partial steroid responsiveness led to diagnostic uncertainty and discontinuation of treatment. Such presentations and non-specific symptoms risk delayed recognition of LVV.

PET-CT was pivotal in establishing the diagnosis. The scan demonstrated widespread aortic and branch-vessel inflammation, including subclavian and carotid involvement, providing a unifying explanation for his severe postural hypotension. Importantly, PET-CT helped distinguish inflammatory uptake from background atherosclerotic calcification seen on CT, a recognised diagnostic challenge in elderly patients.

Treatment required careful balancing of disease control and comorbidity - related risks. High dose steroids resulted in hyperglycaemia requiring short term treatment with insulin. Leflunamide was initiated early because of steroid induced hypoglycaemia and other steroid related comorbidity in this elderly patient.

Monitoring disease activity proved challenging. CRP normalised early despite ongoing symptoms, underscoring the limitations of inflammatory markers in LVV. Functional assessment, weight trends and postural symptoms were more informative. PET-CT provided diagnostic clarity but is not suitable for routine monitoring, reinforcing the need for careful longitudinal clinical evaluation.

This case underscores the need for heightened clinical suspicion of extracranial giant cell arteritis in patients presenting with unexplained postural hypotension and elevated inflammatory markers, even in the absence of typical cranial symptoms. Early recognition may facilitate timely diagnosis and treatment, potentially reducing disease-related morbidity and unnecessary hospital admissions.

**Key learning points:**

**Dizziness and postural hypotension may reflect large vessel involvement** We suggest including giant cell arteritis in the differential diagnosis of unexplained postural hypotension with raised inflammatory markers. Postural dizziness may arise from carotid or vertebral artery involvement in LVV. Recognising this association is essential, as such symptoms are frequently attributed to cardiac, autonomic or metabolic causes rather than vasculitis (Salvarani et al., 2008).

**Atypical systemic presentations of LVV** Large vessel vasculitis can present with non-specific systemic symptoms such as weight loss, anorexia and dizziness, particularly in older adults, and may lack classical cranial GCA or PMR features. Inflammatory markers may be only modestly elevated, contributing to diagnostic delay (Kermani et al., 2012).

**PET-CT is pivotal when conventional imaging is unrevealing** PET-CT is highly valuable in suspected LVV, especially when CT or ultrasound are non-diagnostic. Optimal interpretation requires patients to be on very low dose corticosteroids or ideally off steroids, as glucocorticoids rapidly suppress vascular FDG uptake (Prieto-González et al., 2014). In this case, PET-CT identified widespread aortic and branch-vessel inflammation and PMR-pattern uptake, providing diagnostic clarity.

**Inflammatory markers may normalise early** CRP may normalise despite ongoing symptoms or active disease, limiting its utility in monitoring LVV, as was the case in our patient. Clinical assessment—including functional status, weight trends and symptom evolution—remains central to disease monitoring (Hellmich et al., 2020).

